# Hepatic Steatosis Predicts Higher Incidence of Recurrence in Colorectal Cancer Liver Metastasis Patients

**DOI:** 10.3389/fonc.2021.631943

**Published:** 2021-03-09

**Authors:** Haiyan Chen, Siqi Dai, Yimin Fang, Liubo Chen, Kai Jiang, Qichun Wei, Kefeng Ding

**Affiliations:** ^1^Department of Radiation Oncology, Key Laboratory of Cancer Prevention and Intervention, Ministry of Education, The Second Affiliated Hospital, Zhejiang University School of Medicine, Hangzhou, China; ^2^Zhejiang University Cancer Center, Hangzhou, China; ^3^Department of Colorectal Surgery and Oncology, Key Laboratory of Cancer Prevention and Intervention, Ministry of Education, The Second Affiliated Hospital, Zhejiang University School of Medicine, Hangzhou, China

**Keywords:** colorectal cancer, liver metastasis, hepatic recurrence, hepatic steatosis, L/S ratio

## Abstract

**Purpose:** Colorectal liver metastasis (CRLM) is the major cause of death due to colorectal cancer. Although great efforts have been made in treatment of CRLM, about 60–70% of patients will develop hepatic recurrence. Hepatic steatosis was reported to provide fertile soil for metastasis. However, whether hepatic steatosis predicts higher incidence of CRLM recurrence is not clear. Therefore, we aimed to determine the role of hepatic steatosis in CRLM recurrence in the present study.

**Methods:** Consecutive CRLM patients undergoing curative treatment were retrospectively enrolled and CT liver-spleen attenuation ratio was used to detect the presence of hepatic steatosis. In patients with hepatic steatosis, we also detected the presence of fibrosis. Besides, a systematic literature search was performed to do meta-analysis to further analyze the association between hepatic steatosis and CRLM recurrence.

**Results:** A total of 195 eligible patients were included in our center. Patients with hepatic steatosis had a significantly worse overall (*P* = 0.0049) and hepatic recurrence-free survival (RFS) (*P* = 0.0012). Univariate and multivariate analysis confirmed its essential role in prediction of RFS. Besides, hepatic fibrosis is associated with worse overall RFS (*P* = 0.039) and hepatic RFS (*P* = 0.048). In meta-analysis, we included other four studies, with a total of 1,370 patients in the case group, and 3,735 patients in the control group. The odds ratio was 1.98 (95% CI: 1.25–3.14, *P* = 0.004), indicating that patients with steatosis had a significantly higher incidence of CRLM recurrence.

**Conclusion:** In summary, patients with hepatic steatosis had a significantly worse overall and hepatic RFS and it's associated with higher incidence of CRLM recurrence.

## Introduction

According to GLOBOCAN estimates in 2018, colorectal cancer (CRC) is the third most common type of cancer and the second leading cause of cancer-related deaths globally (1). With great achievements in early tumor detection and multidisciplinary team working, the 5-year survival of CRC increased over time (2, 3). However, colorectal liver metastasis (CRLM) is still the major cause of death due to CRC. About 50% CRC patients will develop liver metastasis over the course of their life (4), and the median survival time is 5–20 months for liver metastasis patients with no treatment (5). Loco-regional treatment of surgical resection and radiofrequency ablation (RFA), and systemic neo-adjuvant treatment has been used to treat liver metastasis (6). However, about 60–70% of patients will develop recurrence, primarily in the liver (7). Therefore, how to prevent recurrence and improve treatment outcomes is one of the most important problems in modern oncology.

Generally, the mechanisms of recurrence are assumed to be inadequate treatment of disease, micro-metastatic dissemination from the primary tumor and awakening of dormant tumor cells (8, 9). In addition to intrinsic recurrent tumor cell phenotype, bidirectional communication between tumor cells and their microenvironment is critical for liver recurrence (10). Liver microenvironment, including cellular and non-cellular components, provides fertile soil for CRC metastasis and recurrence (11). Hepatic steatosis, also known as fatty liver disease, is an accumulation of at least 5% of liver weight fat in liver, and may progress to steatohepatitis and ultimately cirrhosis (12). Prolonged hepatic lipid storage will lead to liver metabolic dysfunction and inflammation (12), which is crucial in establishing a pro-metastatic niche that supports seeding and colonization of metastatic cells (13). However, the association between hepatic steatosis and CRLM recurrence has not been fully addressed. Some studies showed that hepatic steatosis is positively associated with liver metastasis and recurrence of CRC (14, 15), while other studies yielded the contrary results (16, 17). It's imperative to underlie the predictive role of hepatic steatosis in CRLM recurrence, to guide clinicians in making personalized treatment and monitoring strategies for patients with different recurrence risk.

Liver biopsy is the gold standard for diagnosis and grading of hepatic steatosis, but it is invasive and lacks practicality. Computed tomography (CT) provides an accurate and precise quantification of liver fat while also being non-invasive and clinically available (18). Therefore, in this study, we used CT liver-spleen attenuation ratio (L/S ratio) to detect the presence of hepatic steatosis, and identify the association between hepatic steatosis and CRLM recurrence. To overcome the limitation of data from single center and small sample size, we also did a meta-analysis on the basis of multiple centers and a large population to further analyze the association between hepatic steatosis and CRLM recurrence.

## Methods and Materials

### Patients and Treatment

CRLM patients who underwent resection of the primary site and hepatectomy/RFA for liver metastases with curative intention in the Second Affiliated Hospital of Zhejiang University School of Medicine (SAHZU) from June 2012 and December 2019 were consecutively enrolled. All the CRLM patients were histologically diagnosed as colorectal adenocarcinoma, and underwent single-stage or two-stage surgeries with curative intent. All the included patients should achieve no-evidence-of-disease (NED) status by postoperative radiological examinations. All radiological images were reviewed independently by two radiologists and disagreements were resolved through consensus. The exclusion criteria were as follows: (1) without histological diagnosis of colorectal adenocarcinoma, (2) did not achieve status of NED, (3) recurrent CRLM, (4) without routine postoperative surveillance, (5) without non-enhanced pretreatment CT images for steatosis assessment, (6) without active follow up of recurrence date. Recurrence-free survival (RFS) was defined as the number of months between the date of achieving NED and the date of recurrence of any organs (overall RFS) or hepatic recurrence (hepatic RFS) evaluated by radiological examinations. The specific inclusion procedure can be found in the previous study (19). This project was approved by the Independent Ethics Committee of SAHZU and informed consent was obtained from all patients.

### CT Examination and Measurement of CT Attenuation of the Liver and Spleen

Non-enhanced CT images before treatment of liver metastasis were scanned by a second-generation dual-source CT (Statel: SOMATOM Definition AS and Sensation 16, Siemens Medical Solutions, Forchheim, Germany), and reviewed in Picture archiving and communication system (PACS). Two investigators (Haiyan Chen and Siqi Dai) independently extracted the mean CT attenuation values [in Hounsfield units (Hu)] of the region of interests (ROIs) in liver and spleen (Supplementary Figure 1). The ROIs were three same size areas (1-cm circle) in different segment of liver and in the upper, middle and lower thirds of the spleen, avoiding vessels, bile ducts, focal lesions, metastases, and surface lesions. L/S ratio was calculated as mean liver attenuation (Hu)/mean spleen attenuation (Hu) (18, 20). Those with L/S ratio lower than 1.1 was diagnosed with hepatic steatosis (18).

### Calculation of Fibrosis Score

To assess the presence of fibrosis in fatty liver, the aspartate aminotransferase and alanine aminotransferase ratio (AAR) was calculated in patients with hepatic steatosis (21). The serum level of aspartate aminotransferase and alanine aminotransferase was obtained before treatment of liver metastasis. And the cutoff of AAR was obtained by the maxstat package using R version 3.6.1 (R Foundation for Statistical Computing, Vienna, Austria). Patients with AAR >1.08 were defined as fibrotic liver.

### Publication Search and Inclusion for Meta-Analysis

We searched PubMed, MEDLINE, Web of Science, and BIOSIS for articles concerning the association between hepatic steatosis, fibrosis and CRLM recurrence. The last search update was November 2020, using the search terms (“colorectal cancer” or “colorectal carcinoma” or “colorectal tumor” or “colorectal neoplasm” or “colon tumor” or “rectal tumor” or “colon cancer” or “rectal cancer”) AND (“fatty liver” or “hepatic steatosis” or “steatohepatitis” or “NASH” or “fibrosis”) AND (“liver metastasis”) AND (“recurrence”). Additional studies were identified by manual search of the references of the original studies or review articles. All eligible articles were retrieved for titles, abstracts, and full texts. Studies included in our meta-analysis met the following criteria: (1) case-control or case-cohort studies evaluating chronic liver disease of steatosis or fibrosis and CRLM recurrence; (2) contained original data to calculate odds ratios (ORs) and 95% confidence intervals (CIs). The exclusion criteria were as follows: (1) not for CRLM recurrence research; (2) not the chronic liver disease of steatosis or fibrosis; (3) no detailed data of case and control group; and (4) case only or review articles. Besides, Study quality was assessed independently by two authors according to our modified criteria as reported before (22), and the specific scale for quality assessment can be found in Supplementary Table 3. The total scores ranged from 0 to 10, with higher scores indicating better quality.

### Statistics

Pearson *x*^2^-test was employed to investigate significant differences between two groups. Univariate and multivariate models using Cox regression analyses were constructed to evaluate factors correlated with overall and hepatic RFS. The above statistics were performed by SPSS version 19.0 software (SPSS Inc, Chicago, IL). Kaplan-Meier curve was plotted in R version 3.6.1 (R Foundation for Statistical Computing, Vienna, Austria). For meta-analysis, we used STATA version 12.0 (Stata Corporation, College Station, Texas, USA) to quantitatively analyze the impact of liver disease on CRC liver recurrence risk. The specific statistical methods can been found in the previous study (22). A *P* < 0.05 was statistically significant. All tests were two sided, and 95% CIs were used.

## Results

### Characteristics of the Included Patients With or Without Hepatic Steatosis by the CT Images

**Figure 1:**
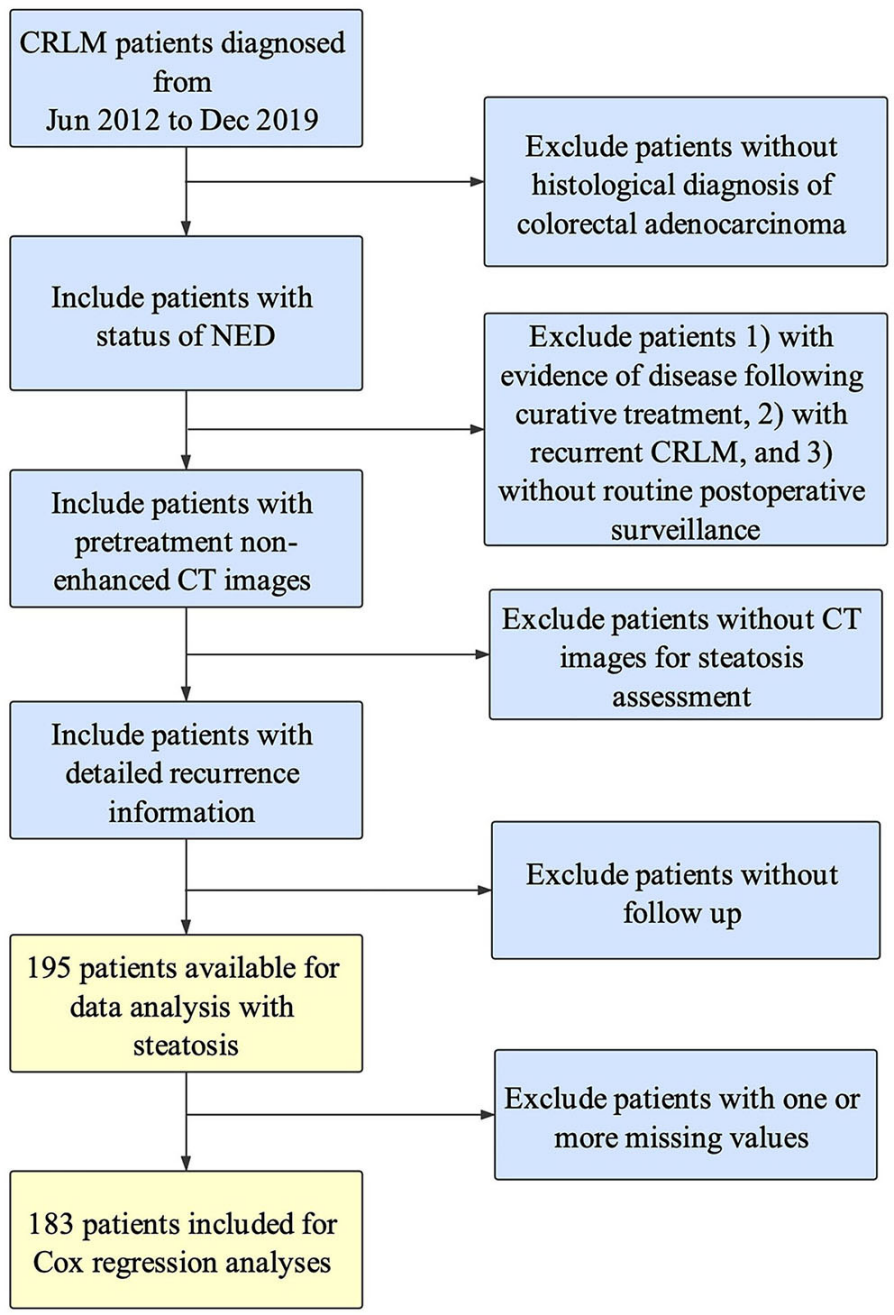
Flow chart of patient inclusion.

A total of 195 patients were assessed as eligible for inclusion in this study by using the patient selection algorithm described in the Methods section (Figure 1), among which 39 (20.00%) were diagnosed with hepatic steatosis by CT images with L/S ratio ≤1.1. The clinicopathological characteristics of these 195 patients and the association with hepatic steatosis were shown in Table 1. There were 49.23% patients (*N* = 96) <60 years old, and 50.77% patients (*N* = 99) more than 60 years old. Thirty-one percent patients (*N* = 61) were female, and 68.72% (*N* = 134) were male. BMI higher than 25 was observed in 21.03% (*N* = 41) of patients, and 12.31% (*N* = 24) of patients were diagnosed with Diabetes Mellitus. Referring to the primary tumor, 66.15% (*N* = 129) were located in colon, and the rest (32.31%, *N* = 63) in the rectum. Besides, 38.97% of them (*N* = 76) were pathologically diagnosed as T4, with tumor extension through the serosa. There were 33.85% of patients (*N* = 66) without regional lymph node metastasis, 33.33% (*N* = 65) with metastasis in 1–3 regional lymph nodes, and 27.69% (*N* = 54) with metastasis in 4 or more regional lymph nodes. Regarding treatment, 57.95% (*N* = 113) and 71.79% (*N* = 140) of patients received preoperative and postoperative chemotherapy, respectively. Fifty-two percent patients (*N* = 103) underwent curative hepatectomy for CRLM, while 30.77% (*N* = 60) of patients underwent RFA, and the rest (*N* = 32) received both hepatectomy and RFA. Ten percent patients (*N* = 20) had more than 5 liver metastases, and 20.00% of patients (*N* = 39) had liver metastasis more than 5 cm. Besides, KRAS and BRAF mutation was found in 18.97% (*N* = 37) and 1.54% (*N* = 3) of patients, but 47.18 and 50.26% of patients did not have KRAS and BRAF mutation results. For these included parameters, there were no statistically significant differences between patients with and without steatosis.

**Table 1:** Clinical and pathological characteristics of included patients with or without hepatic steatosis.

	**Total patients**		**With hepatic steatosis**		**Without hepatic steatosis**		***P*-value^†^**
**Characteristics**	***N***	**%**	***N***	**%**	***N***	**%**	
**Total**	195	100.00	39	100.00	156	100	
**Age at diagnosis**							0.086
≤60	96	49.23	24	61.54	72	46.15	
>60	99	50.77	15	38.46	84	53.85	
**Sex**							0.757
Male	134	68.72	26	66.67	108	69.23	
Female	61	31.28	13	33.33	48	30.77	
**BMI**							0.934
≤25	154	78.97	33	84.62	121	77.56	
>25	41	21.03	6	15.38	35	22.44	
**Diabetes mellitus**							0.327
Without	171	87.69	36	92.31	135	86.54	
With	24	12.31	3	7.69	21	13.46	
**Primary tumor location**							0.938
Colon	129	66.15	26	66.67	103	66.03	
Rectum	63	32.31	13	33.33	50	32.05	
**Depth of tumor invasion**							0.607
≤T3	109	55.90	21	53.85	88	56.41	
T4	76	38.97	17	43.59	59	37.82	
**Lymph node stage**							0.259
N0	66	33.85	9	23.08	57	36.54	
N1	65	33.33	16	41.03	49	31.41	
N2	54	27.69	12	30.77	42	26.92	
**Maximum size of liver metastases**							0.591
≤5 cm	156	80.00	30	76.92	126	80.77	
>5 cm	39	20.00	9	23.08	30	19.23	
**Number of liver metastasis**							0.238
≤5	175	89.74	33	84.62	142	91.03	
>5	20	10.26	6	15.38	14	8.97	
**Preoperative chemotherapy**							0.885
No	82	42.05	16	41.03	66	42.31	
Yes	113	57.95	23	58.97	90	57.69	
**Postoperative chemotherapy**							0.948
No	53	27.18	10	25.64	43	27.56	
Yes	140	71.79	27	69.23	113	72.44	
**Surgery type**							0.639
Hepatectomy	103	52.82	18	46.15	85	54.49	
Radiofrequency ablation	60	30.77	14	35.90	46	29.49	
Hepatectomy+Radiofrequency ablation	32	16.41	7	17.95	25	16.03	
**KRAS mutation**							0.547
No	66	33.85	16	41.03	50	32.05	
Yes	37	18.97	6	15.38	31	19.87	
Unknown	92	47.18	17	43.59	75	48.08	
**BRAF mutation**							0.632
No	94	48.21	18	46.15	76	48.72	
Yes	3	1.54	0	0.00	3	1.92	
Unknown	98	50.26	21	53.85	77	49.36	

† *Pearson x^2^-test*.

### Hepatic Steatosis Is a Predictor of Overall and Hepatic RFS of CRLM Patients

**Figure 2:**
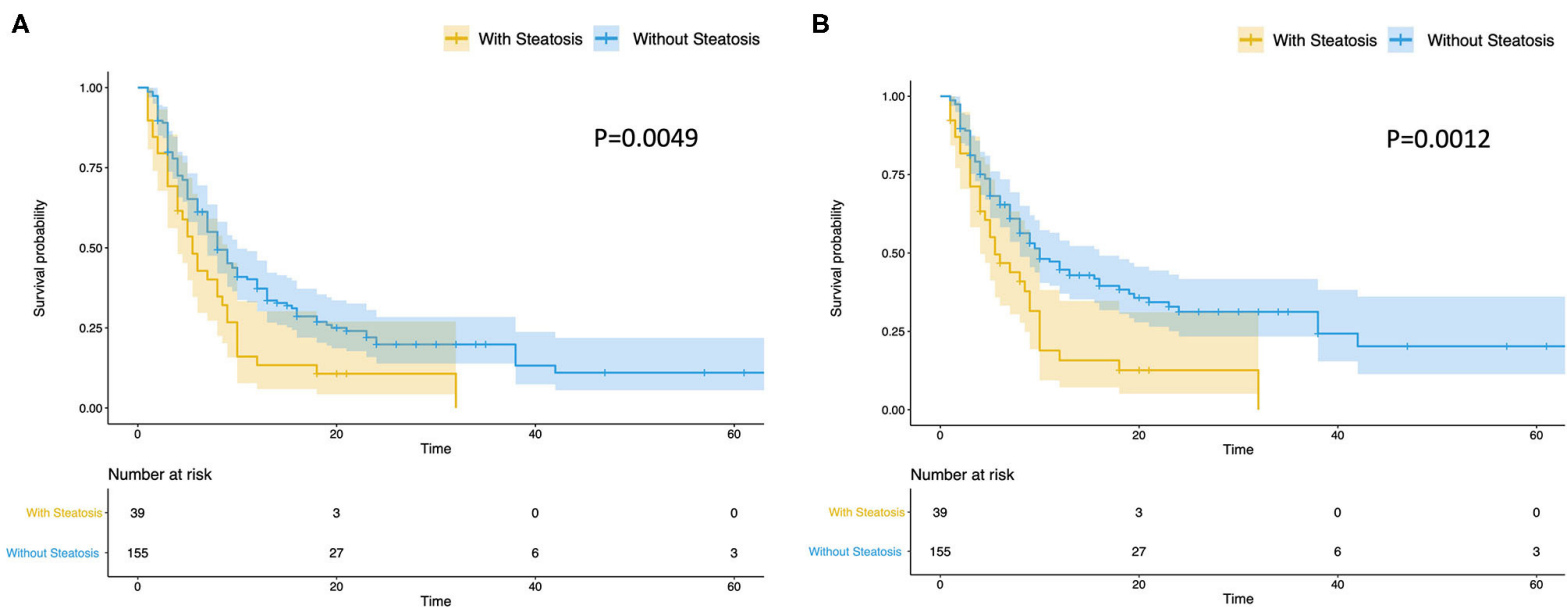
Hepatic steatosis is a predictor of overall and hepatic RFS of CRLM. Patients with steatosis (*N* = 39) had a significantly worse overall RFS **(A)** (*P* = 0.0049) and hepatic RFS **(B)** (*P* = 0.0012) than Patients without steatosis (*N* = 156).

The terminal event of our follow-up was cancer recurrence, regardless of organs, and the median follow-up period for participants included was 7.0 months (IQR: 3.5–14.0 months). Recurrence of any organ was observed in 153 (78.46%) of 195 patients. There were 124 patients with hepatic recurrence, among which 88 (70.97%) had liver-only recurrence, and the other 36 (29.03%) had multiple organ recurrences. In patients with hepatic steatosis, hepatic recurrence was observed in 82.05% of patients (32/39), while the recurrence rate was 58.97% (92/156) in patients without steatosis. As shown in Figure 2, patients with hepatic steatosis had a significantly worse overall RFS (*P* = 0.0049) and hepatic RFS (*P* = 0.0012). For extrahepatic RFS, no significant difference was found in these two groups (*P* = 0.68). Besides, Cox regression analyses confirmed the role of hepatic steatosis in prediction of overall RFS (HR = 1.86, 95% CIs: 1.23–2.82, *P* = 0.003) (Figure 4A and Supplementary Table 1) and hepatic RFS (HR = 2.07, 95% CIs: 1.33–3.22, *P* = 0.001) (Figure 4B and Supplementary Table 2) in CRLM patients. In addition to hepatic steatosis, number of liver metastasis, preoperative chemotherapy, and KRAS mutation were also identified as significant predictors of hepatic RFS (Figure 4B and Supplementary Table 2).

### Hepatic Fibrosis Is Associated With Worse Overall and Hepatic RFS in Patients With Hepatic Steatosis

**Figure 3:**
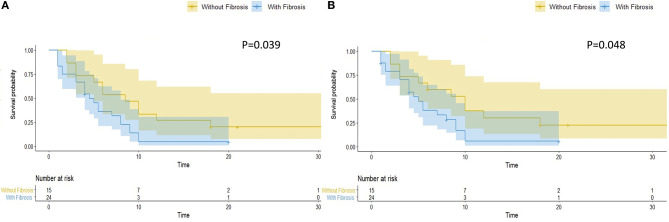
Hepatic fibrosis is associated with worse overall and hepatic RFS in patients with hepatic steatosis. In patients with hepatic steatosis, patients with fibrosis (*N* = 24) had a significantly worse overall RFS **(A)** (*P* = 0.039) and hepatic RFS **(B)** (*P* = 0.048) than Patients without fibrosis (*N* = 15).

Hepatic fibrosis is the key pathological feature of progressive liver disease and is a prognostic factor for the development of hepatic Steatosis (23). We evaluated hepatic fibrosis in patients with steatosis by AAR, which is a non-invasive blood marker (21). We divided patients with hepatic steatosis into two groups, with (*N* = 24, 61.5%) and without (*N* = 15, 38.5%) hepatic fibrosis. Patients with hepatic fibrosis had a significantly worse overall RFS (*P* = 0.039) (Figure 3A) and hepatic RFS (*P* = 0.048) (Figure 3B). For extrahepatic RFS, no significant difference was found in these two groups (*P* = 0.58).

**Figure 4:**
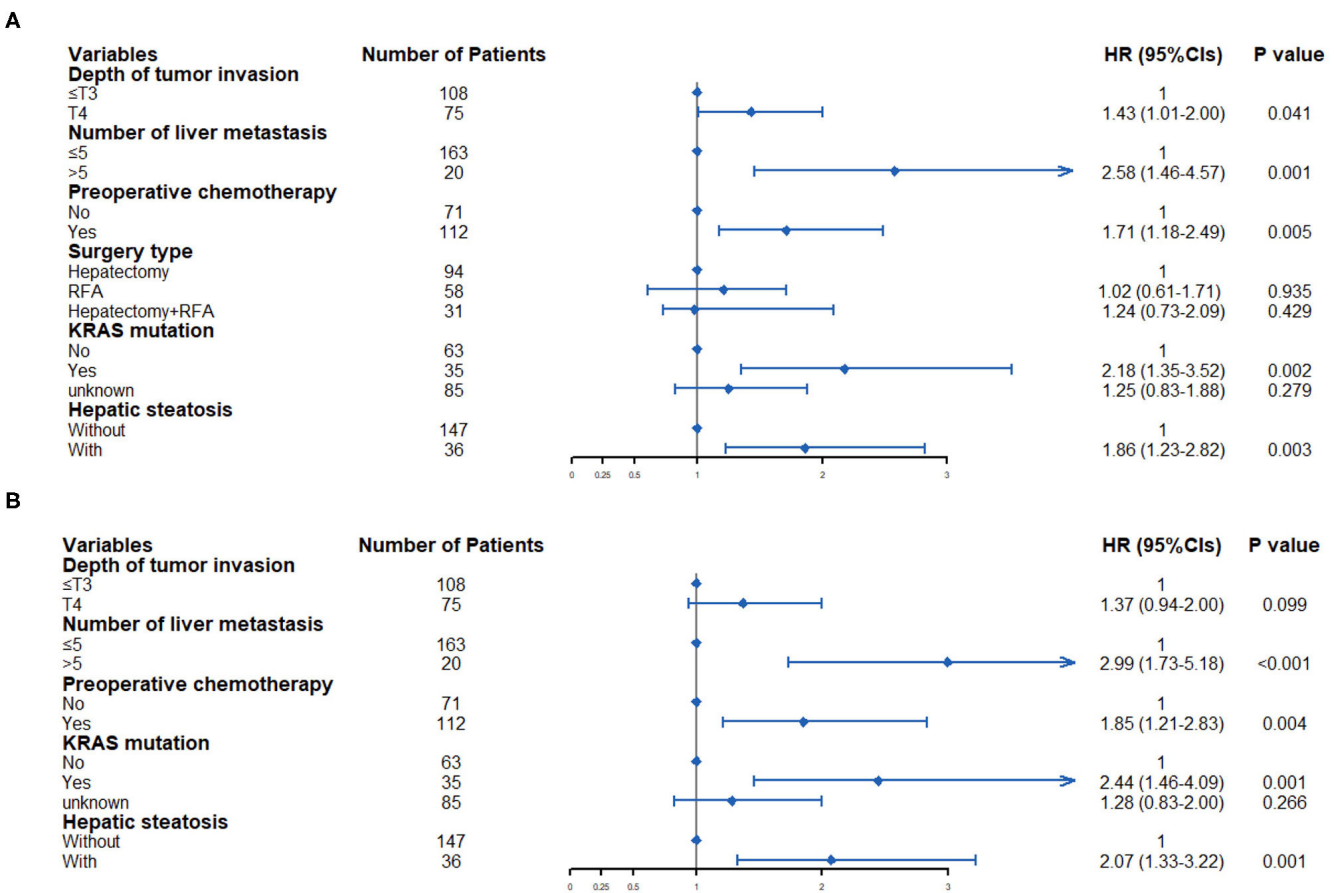
Multivariate Cox regression analysis was used to determine predictive factors for overall RFS **(A)** and hepatic RFS **(B)** in CRLM patients.

### Hepatic Steatosis and Fibrosis Is Associated With Higher Risk of CRLM Recurrence Analyzed by a Meta-Analysis

**Figure 5:**
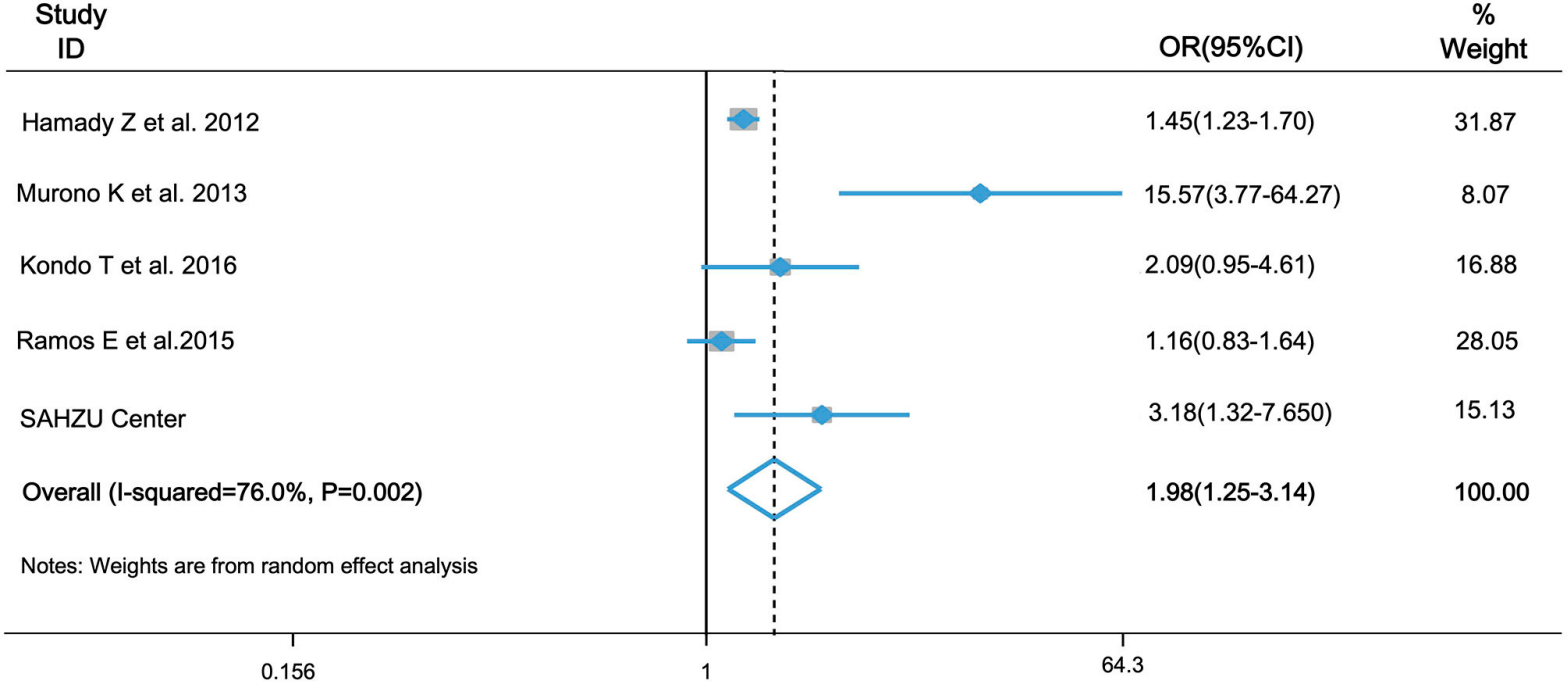
Forest plot of OR for association of hepatic steatosis and fibrosis and risk of CRLM recurrence by meta-analysis. OR was 1.98 (95% CI: 1.25–3.143, *P* = 0.004) in the random-effects model by meta-analysis. It indicated that hepatic steatosis and fibrosis is associated with higher risk of CRLM recurrence.

During the past 10 years, several studies have reported the association of hepatic steatosis and recurrence of CRLM. However, the clinical use of hepatic steatosis as a risk factor of CRLM recurrence is still under doubt and the results are inconsistent. Therefore, we did a meta-analysis to determine the role of hepatic steatosis in hepatic recurrence of CRLM. There were four eligible articles included, and the characteristics of chosen articles are summarized in Table 2. Liver disease in three of them was steatosis by histologic assessment or CT images, and the other one was hepatic fibrosis by serum fibrosis score. Study quality score ranges from 7 to 10 (Table 2). The studies were conducted in UK, Spain and Japan. Combined with our study, there were totally 1,370 patients in the case group, and 3,735 patients in the control group. As Figure 5 showed, there was heterogeneity among the studies (*I*_2_= 76.0%, *P* = 0.002) and then random-effects model was adopted. The odds ratio, expressed as liver disease group vs. normal liver group, was 1.98 (95% CI: 1.25–3.14, *P* = 0.004, random-effects model). This result demonstrated that patients with steatosis or fibrosis had a significantly higher incidence of CRLM recurrence than those with normal livers. Egger's (Supplementary Figure 2A) and Begg's test (Supplementary Figure 2B) was done to estimate the publication bias of literatures. No evidence of publication bias was observed, with *P*-value of 0.09 and 0.15, respectively.

**Table 2:** Characteristics of studies included in the meta-analysis.

**ID**	**Study**	**Country**	**Quality score**	**Sample size(case/control)**	**Liver disease**	**Diagnosis method**	**References**
1	Hamady et al. (2013)	UK	10	927/1,788	Steatosis	Histologic diagnosis	(14)
2	Kondo et al. (2016)	Japan	8	77/876	Fibrosis	Serum NAFLD fibrosis score	(24)
3	Ramos et al. (2016)	Spain	9	264/264	Steatosis	Histologic diagnosis	(17)
4	Murono et al. (2013)	Japan	7	63/651	Steatosis	CT liver-spleen ratio	(16)
5	SAHZU center	China	7	39/156	Steatosis	CT liver-spleen ratio	/

## Discussion

In this study, we used L/S ratio to evaluate the presence of hepatic steatosis. The HU attenuation of liver extracted from CT images is usually higher than the spleen. When L/S ratio is reversed and <1.1, the patient can be diagnosed with hepatic steatosis (25). Although the sensitivity of L/S ratio is not very high, it's still the widely used approach to diagnose hepatic steatosis, due to its non-invasive nature and easy access (26). Liver biopsy is the gold standard for hepatic steatosis, but it is invasive with a risk of complications and evaluates only a very small portion of the overall organ (27). It will cause sampling error because of heterogeneous development in chronic liver disease (28). Liver tissues of some studies come from surgical resection specimens, and microscopic analysis was done by examination of the non-cancerous part of the surgical specimen (14). Although normal tissue adjacent to the tumor is usually used as a normal control in cancer studies, transcriptome analysis showed that it is a unique intermediate state between healthy and tumor tissue (29). Tissue surrounding the tumor in surgical specimen will be edited by the tumor itself, and may not be normal (29). Therefore, in this study, we still used L/S ratio to detect hepatic steatosis, which will evaluate a greater volume of liver parenchyma than biopsy (28).

Univariate and multivariate analysis showed that patients with hepatic steatosis had a significantly worse overall and hepatic RFS, but not extrahepatic RFS. It is consistent with the result from Hamady et al. (14) that hepatic steatosis was an independent predictor of local hepatic recurrence following resection of CRLM, in which hepatic steatosis was identified by microscopic analysis. However, Ramos et al. (17) showed that hepatic steatosis was not significantly associated with CRLM liver recurrence after resection. Considering that these studies did not yield consistent results, we did a meta-analysis to determine the role of hepatic steatosis in hepatic recurrence of CRLM. Meta-analysis is a statistical method that combines results from a series of studies and thousands of patients (30, 31). It reduces the risk of false-negative results and increases statistical power by increasing sample size, and can identify subgroups with most significant effects by combining data from numerous studies (30, 31). Mata-analysis will be a good statistical approach to resolve the limitation of one-single center data and small sample size in our study. By analysis of data from our center, and meta-analysis of 1,370 and 3,735 patients in the case and control group, respectively, we concluded that hepatic steatosis significantly predicts higher CRLM recurrence. Statins are effective in the management of hypercholesterolemia, and can reduce the development of hepatic steatosis (32). Two large population-based cohorts showed that statin use after diagnosis of CRC was associated with reduced cancer-related mortality, and longer overall survival (33, 34). These results suggested that hepatic steatosis increased the incidence of CRLM recurrence, implying the need of a proper nutrition and lifestyle in colorectal cancer survivors (35–37).

In animal study, high fat diet-induced steatosis profoundly increase liver metastasis in a splenic injection model of experimental liver metastasis using syngeneic MC38 colon cancer cells (38). Referring to potential mechanisms that contribute to CRLM, Li et al. (39) found that mice with hepatic steatosis have a marked predisposition to liver metastasis, which is unusual in non-steatotic mice. The mechanism is that lipolytic products are transferred to cancer cells via fatty acid transporter protein 1, and promote cancer cells growth by mitochondrial oxidation (39). Hepatic steatosis can cause extracellular matrix (ECM) remodeling and reorganization, which create a fibrotic niche for CRLM and is important in tumor promotion and growth (40). Transforming growth factor β (TGF-β), which participants in pathogenesis of hepatic steatosis, is pivotal in maintaining liver homeostasis and have a leading role in CRLM (41). In addition to TGF-β, other cytokines, such as IL-1, IL-6, and TNF-α, contribute significantly to the pathophysiology of hepatic steatosis through stimulation of hepatic inflammation, and can in turn promote CRLM (42). Dysregulated cytokines and chemokines can recruit a variety of regulatory and suppressive immune cells to establish the pre-metastatic niche for CRLM (43). For example, Zhang et al. (44) found that IL-33, an IL-1 cytokine family member, promotes CRLM by modulating the tumor microenvironment. Besides, Tiwary et al. found that lipid metabolic profile directly affect immune-modulatory function of NKT cells, which have influence on anti-tumor immunity in turn (45). Overall, hepatic Steatosis establishes a favorable microenvironment for tumor seeding. Further studies should be conducted to explore the molecular mechanisms by which hepatic steatosis promotes CRLM recurrence.

Non-alcoholic fatty liver disease (NAFLD) is the buildup of extra fat in liver cells that is not caused by alcohol, including hepatic steatosis and non-alcoholic steatohepatitis, with varying amounts of advanced fibrosis and cirrhosi s (46, 47). The majority of NAFLD has simple steatosis, and about 10–30% develops steatohepatitis, and ultimately cirrhosis. As the initial and critical step for the pathogenesis of steatohepatitis and cirrhosis (12), hepatic fibrosis results from an imbalance between the new deposition and desorption of ECM (46). Hepatic fibrosis has been reported to be the most important prognostic factor for the development of liver disease. The gold standard for fibrosis is histological assessment of liver, but it is invasive and lacks routine clinical use (23). Blood markers, such as AST-to-platelet ratio index, tissue inhibitor of metalloproteinase-1, Collagen type IV, and AAR have been proposed for the assessment of liver fibrosis (48). In this study, we used serum-based AAR to evaluate the presence of fibrosis in patients with hepatic steatosis. We demonstrated that in patients with hepatic steatosis, patients with hepatic fibrosis had a significantly worse overall and hepatic RFS, but not extrahepatic RFS. This result is consistent with the finding of other groups that the hepatic fibrosis is associated with worse outcomes (24, 49). Besides, a prospective study showed that hepatic steatosis and fibrosis had a highly significant shared gene effect of 0.756, and genes involved with steatosis pathogenesis may also be involved with fibrosis pathogenesis (49). Patients with genetic susceptibility to hepatic steatosis also have genetic susceptibility to hepatic fibrosis (49). Considering the close interaction between hepatic steatosis and fibrosis, in further meta-analysis, we evaluated the association between hepatic steatosis and fibrosis and recurrence of CRLM. In this study, limited by the data availability, we used only AAR to evaluate fibrosis in this study, without histology confirmation. Although it is non-invasive, blood markers cannot achieve satisfactory accuracy and complete validation of hepatic fibrosis in some cases (48). Therefore, it requires further studies with histological assessment to validate the association of hepatic fibrosis and CRLM.

In addition to hepatic steatosis, we also found that depth of tumor invasion, number of liver metastasis, preoperative chemotherapy and KRAS mutation was significantly associated with overall RFS. Referring to hepatic RFS, number of liver metastasis, preoperative chemotherapy, and KRAS mutation were identified as significant predictors. Those were consistent with other reported studies (50, 51). In our study, mutation information of KRAS and BRAF was only available in 50% of patients, partly because that the detection method was cost-prohibitive and not covered by basic medical insurance in China. Besides, the gene mutation information in this study was derived from the primary tumor, not the metastatic sites. Considering the genetic evolution and alterations from primary to matched metastatic tissues (52, 53), it is interesting to investigate the association between gene mutation in metastatic tissues and CRLM recurrence in the future.

Undoubtedly, limitations existed in this study. First, the diagnosis of hepatic steatosis and fibrosis by L/S ratio and AAR is not the gold standard. Second, this was a single institute based study, without multi-institutional validation. Third, longer follow-up is needed. Third, longer follow-up is needed. Additionally, meta-analysis partially resolves the above limitations, but we have to deny the potential heterogeneity and publication bias among included studies, which might contribute to potential false positivity (22).

In summary, we identified that patients with hepatic steatosis had a significantly worse overall and hepatic RFS in our center. Further meta-analysis showed that hepatic steatosis is associated with higher incidence of CRLM recurrence. Future studies are warranted to investigate the underlying mechanisms by which hepatic steatosis regulates liver metastasis and recurrence, and the potential role of it in CRLM recurrence prevention and treatment.

## Data Availability Statement

The raw data supporting the conclusions of this article will be made available by the authors, without undue reservation.

## Ethics Statement

The studies involving human participants were reviewed and approved by Ethics Committee of Second Affiliated Hospital of Zhejiang University School of Medicine. The patients/participants provided their written informed consent to participate in this study.

## Author Contributions

KD designed the study. YF, LC, and KJ collected patient data. HC and SD analyzed and performed the statistical analysis. HC wrote the manuscript, and QW helped to modify it. All authors read and approved the final manuscript.

## Conflict of Interest

The authors declare that the research was conducted in the absence of any commercial or financial relationships that could be construed as a potential conflict of interest.

## Supplementary Material

Supplementary Figure 1 Representative CT images of liver with **(A)** and without **(B)** hepatic steatosis, evaluated by L/S ratio.

Supplementary Figure 2 Egger's publication bias plot **(A)** and Begg's funnel plot **(B)** showed low risk of publication bias in meta-analysis, with *P*-value of 0.09 and 0.15, respectively.

Supplementary Table 1 Risk factors for overall RFS by univariate and multivariate Cox regression analysis.

Supplementary Table 2 Risk factors for hepatic RFS by univariate and multivariate Cox regression analysis.

Supplementary Table 3 Scale for quality assessment of included studies for meta-analysis.
