# Corrigendum: Hepatic Steatosis Predicts Higher Incidence of Recurrence in Colorectal Cancer Liver Metastasis Patients

**DOI:** 10.3389/fonc.2021.742987

**Published:** 2021-08-18

**Authors:** Haiyan Chen, Siqi Dai, Yimin Fang, Liubo Chen, Kai Jiang, Qichun Wei, Kefeng Ding

**Affiliations:** ^1^Department of Radiation Oncology, Key Laboratory of Cancer Prevention and Intervention, Ministry of Education, The Second Affiliated Hospital, Zhejiang University School of Medicine, Hangzhou, China; ^2^Zhejiang University Cancer Center, Hangzhou, China; ^3^Department of Colorectal Surgery and Oncology, Key Laboratory of Cancer Prevention and Intervention, Ministry of Education, The Second Affiliated Hospital, Zhejiang University School of Medicine, Hangzhou, China

**Keywords:** colorectal cancer, liver metastasis, hepatic recurrence, hepatic steatosis, L/S ratio

In the original article, there was a mistake in **Figure 3** as published. The text in **Figure 3** should be changed into “without fibrosis” and “with fibrosis,” rather than “without steatosis” and “with steatosis.” The corrected **Figure 3** appears below.

**Figure 3:**
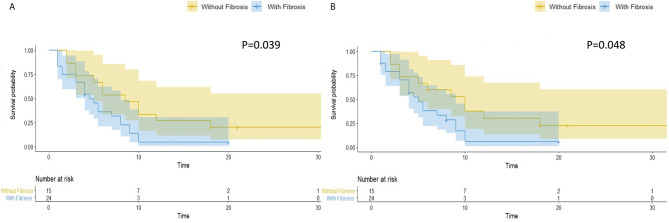
Hepatic fibrosis is associated with worse overall and hepatic RFS in patients with hepatic steatosis. In patients with hepatic steatosis, patients with fibrosis (N = 24) had a significantly worse overall RFS **(A)** (P = 0.039) and hepatic RFS **(B)** (P = 0.048) than Patients without fibrosis (N = 15).

In the original article, there was a typo error in the *Materials and Methods* section. It should be *Supplementary Table 3*, rather than *Supplementary Table 1* in *Publication Search and Inclusion for Meta-Analysis* subsection.

The correction has been made to *Materials and Methods*, *Publication Search and Inclusion for Meta-Analysis*:

We searched PubMed, MEDLINE, Web of Science, and BIOSIS for articles concerning the association between hepatic steatosis, fibrosis and CRLM recurrence. The last search update was November 2020, using the search terms (“colorectal cancer” or “colorectal carcinoma” or “colorectal tumor” or “colorectal neoplasm” or “colon tumor” or “rectal tumor” or “colon cancer” or “rectal cancer”) AND (“fatty liver” or “hepatic steatosis” or “steatohepatitis” or “NASH” or “fibrosis”) AND (“liver metastasis”) AND (“recurrence”). Additional studies were identified by manual search of the references of the original studies or review articles. All eligible articles were retrieved for titles, abstracts, and full texts. Studies included in our meta-analysis met the following criteria: (1) case-control or case-cohort studies evaluating chronic liver disease of steatosis or fibrosis and CRLM recurrence; (2) contained original data to calculate odds ratios (ORs) and 95% confidence intervals (CIs). The exclusion criteria were as follows: (1) not for CRLM recurrence research; (2) not the chronic liver disease of steatosis or fibrosis; (3) no detailed data of case and control group; and (4) case only or review articles. Besides, Study quality was assessed independently by two authors according to our modified criteria as reported before (22), and the specific scale for quality assessment can be found in **Supplementary Table 3**. The total scores ranged from 0 to 10, with higher scores indicating better quality.

Besides, the **Figure 3** and **Figure 4** are misplaced and incorrectly mentioned in the *Results* section. It should be **Figure 4** in subsection *Hepatic steatosis is a predictor of overall and hepatic RFS of CRLM patients*, and **Figure 3** in subsection *Hepatic fibrosis is associated with worse overall and hepatic RFS in patients with hepatic steatosis*.

The correction has been made to *Results*, *Hepatic steatosis is a predictor of overall and hepatic RFS of CRLM patients*:

The terminal event of our follow-up was cancer recurrence, regardless of organs, and the median follow-up period for participants included was 7.0 months (IQR: 3.5–14.0 months). Recurrence of any organ was observed in 153 (78.46%) of 195 patients. There were 124 patients with hepatic recurrence, among which 88 (70.97%) had liver-only recurrence, and the other 36 (29.03%) had multiple organ recurrences. In patients with hepatic steatosis, hepatic recurrence was observed in 82.05% of patients (32/39), while the recurrence rate was 58.97% (92/156) in patients without steatosis. As shown in Figure 2, patients with hepatic steatosis had a significantly worse overall RFS (*P* = 0.0049) and hepatic RFS (*P* = 0.0012). For extrahepatic RFS, no significant difference was found in these two groups (*P* = 0.68). Besides, Cox regression analyses confirmed the role of hepatic steatosis in prediction of overall RFS (HR = 1.86, 95% CIs: 1.23–2.82, *P* = 0.003) (**Figure 4A** and **Supplementary Table 1**) and hepatic RFS (HR = 2.07, 95% CIs: 1.33–3.22, *P* = 0.001) (**Figure 4B** and **Supplementary Table 2**) in CRLM patients. In addition to hepatic steatosis, number of liver metastasis, preoperative chemotherapy, and KRAS mutation were also identified as significant predictors of hepatic RFS (**Figure 4B** and **Supplementary Table 2**).

The correction has also been made to *Results*, *Hepatic fibrosis is associated with worse overall and hepatic RFS in patients with hepatic steatosis*:

Hepatic fibrosis is the key pathological feature of progressive liver disease and is a prognostic factor for the development of hepatic Steatosis (23). We evaluated hepatic fibrosis in patients with steatosis by AAR, which is a non-invasive blood marker (21). We divided patients with hepatic steatosis into two groups, with (*N* = 24, 61.5%) and without (*N* = 15, 38.5%) hepatic fibrosis. Patients with hepatic fibrosis had a significantly worse overall RFS (*P* = 0.039) (**Figure 3A**) and hepatic RFS (*P* = 0.048) (**Figure 3B**). For extrahepatic RFS, no significant difference was found in these two groups (*P* = 0.58).

The authors apologize for these errors and state that this does not change the scientific conclusions of the article in any way. The original article has been updated.

## Publisher’s Note

All claims expressed in this article are solely those of the authors and do not necessarily represent those of their affiliated organizations, or those of the publisher, the editors and the reviewers. Any product that may be evaluated in this article, or claim that may be made by its manufacturer, is not guaranteed or endorsed by the publisher.

