# Supplementary figures and images for: Hepatic Steatosis Predicts Higher Incidence of Recurrence in Colorectal Cancer Liver Metastasis Patients
Source: Front Oncol. 2021 Mar 9;11:631943. doi: 10.3389/fonc.2021.631943 (PMC7986714; doi:10.3389/fonc.2021.631943)

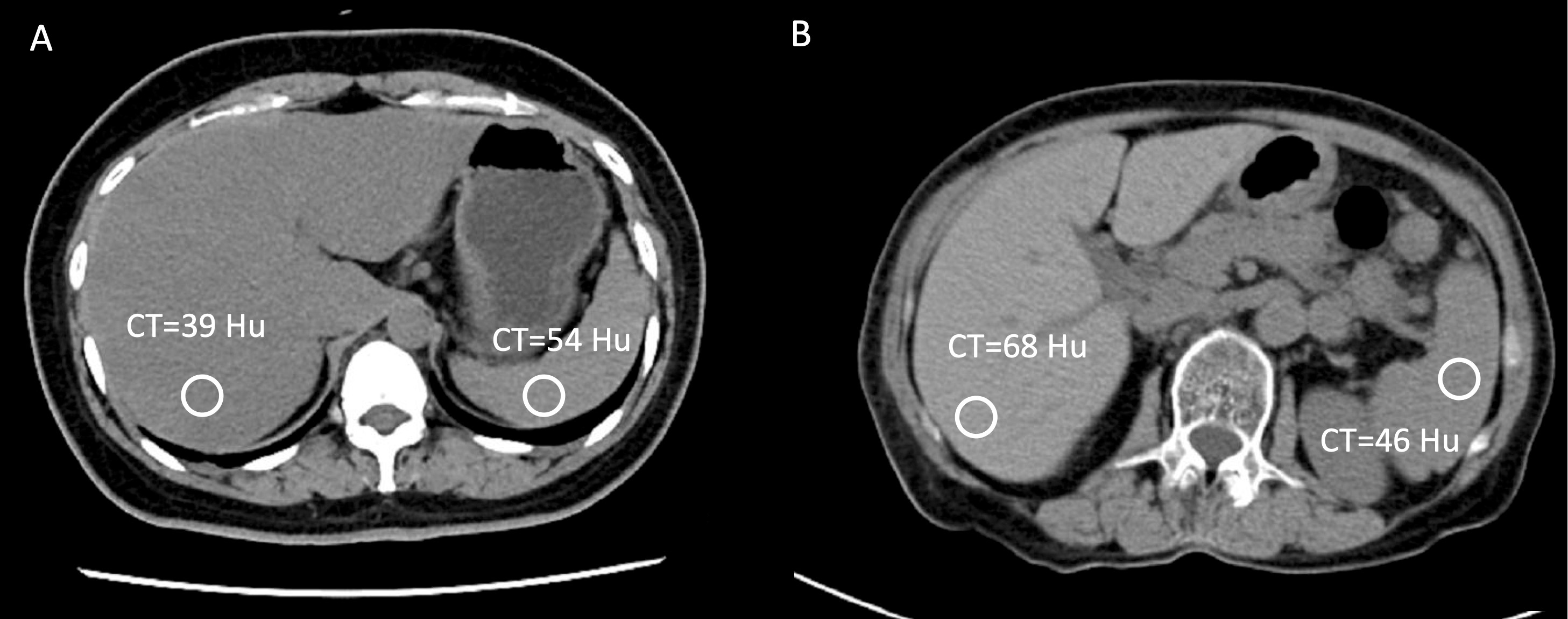

Supplement: Supplementary Figure 1 — Representative CT images of liver with (A) and without (B) hepatic steatosis, evaluated by L/S ratio. [file Image_1.JPEG]

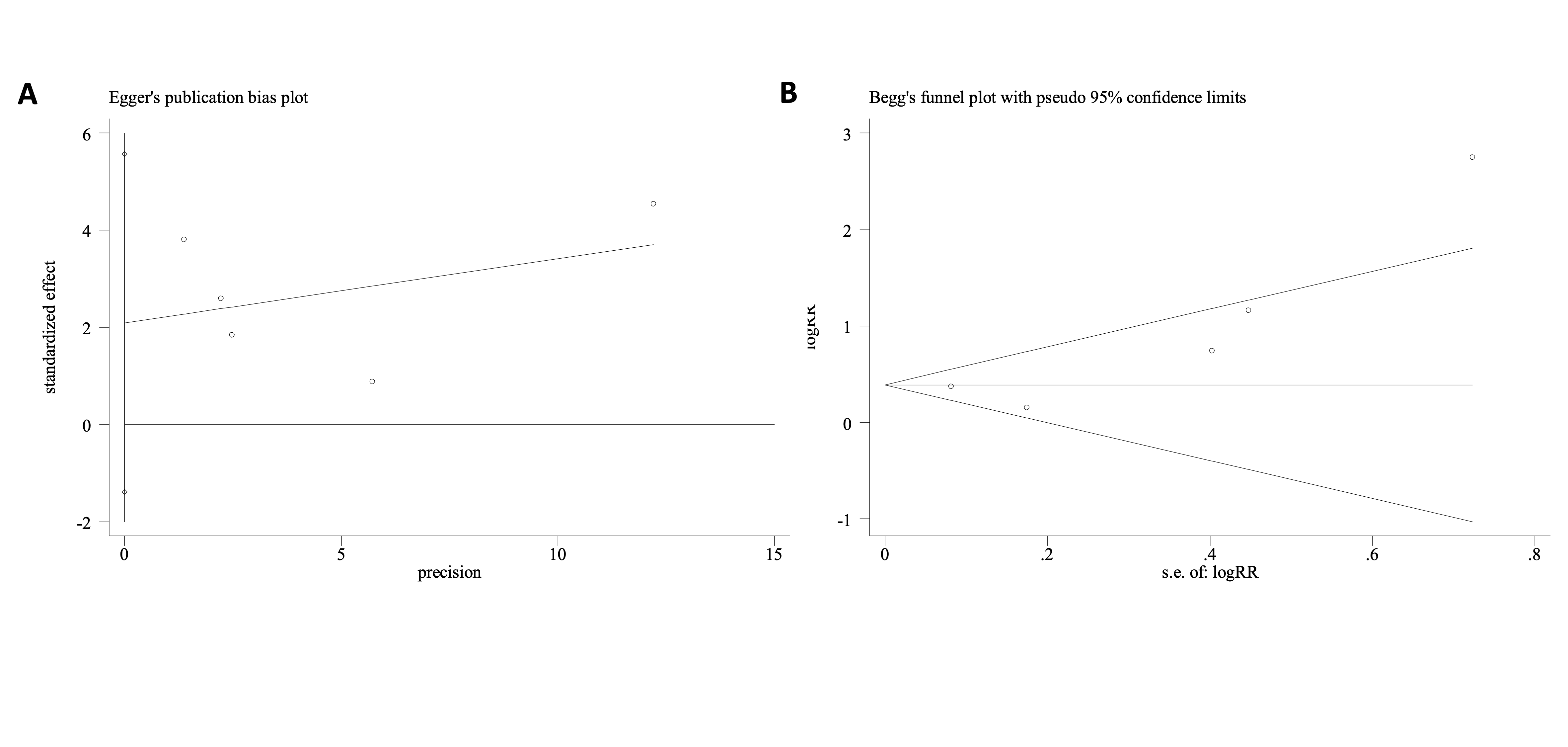

Supplement: Supplementary Figure 2 — Egger's publication bias plot (A) and Begg's funnel plot (B) showed low risk of publication bias in meta-analysis, with P-value of 0.09 and 0.15, respectively. [file Image_2.PNG]
