# Supplementary material for: Hepatic Steatosis Predicts Higher Incidence of Recurrence in Colorectal Cancer Liver Metastasis Patients
Source: Front Oncol. 2021 Mar 9;11:631943. doi: 10.3389/fonc.2021.631943 (PMC7986714; doi:10.3389/fonc.2021.631943)
Supplement: Supplementary Table 1 — Risk factors for overall RFS by univariate and multivariate Cox regression analysis. [file Table_1.DOCX]

**Table S1. Risk factors for overall RFS by univariate and multivariate analysis**

|  | **Univariate analysis** | | | **Multivariate analysis** | | |
| --- | --- | --- | --- | --- | --- | --- |
| **Variables** | **HR** | **95% CIs** | **P** | **HR** | **95% CIs** | **P** |
| **Total** |  |  |  |  |  |  |
| **Age at diagnosis** |  |  | 0.74 |  |  |  |
| ≤60 | 1 |  |  |  |  |  |
| >60 | 0.95 | 0.68-1.31 |  |  |  |  |
| **Sex** |  |  | 0.56 |  |  |  |
| Male | 1 |  |  |  |  |  |
| Female | 0.9 | 0.62-1.29 |  |  |  |  |
| **BMI** |  |  | 0.47 |  |  |  |
| ≤25 | 1 |  |  |  |  |  |
| >25 | 1.16 | 0.78-1.73 |  |  |  |  |
| **Diabetes Mellitus** |  |  | 0.634 |  |  |  |
| Without | 1 |  |  |  |  |  |
| With | 1.13 | 0.69-1.83 |  |  |  |  |
| **Primary tumor location** |  |  | 0.159 |  |  |  |
| Colon | 1 |  |  |  |  |  |
| Rectum | 0.78 | 0.55-1.11 |  |  |  |  |
| **Depth of tumor invasion** |  |  | 0.006 |  |  |  |
| ≤T3 | 1 |  |  | 1 |  |  |
| T4 | 1.6 | 1.15-2.23 |  | 1.43 | 1.01-2.00 | 0.041 |
| **Lymph node stage** |  |  | 0.473 |  |  |  |
| N0 | 1 |  |  |  |  |  |
| N1 | 1.15 | 0.78-1.69 | 0.496 |  |  |  |
| N2 | 1.29 | 0.86-1.95 | 0.223 |  |  |  |
| **Maximum size of liver metastases** |  |  | 0.238 |  |  |  |
| ≤5 cm | 1 |  |  |  |  |  |
| >5 cm | 1.28 | 0.85-1.91 |  |  |  |  |
| **Number of liver metastasis** |  |  | <0.001 |  |  |  |
| ≤5 | 1 |  |  | 1 |  |  |
| >5 | 2.9 | 1.77-4.74 |  | 2.58 | 1.46-4.57 | 0.001 |
| **Preoperative chemotherapy** |  |  | <0.001 |  |  |  |
| No | 1 |  |  | 1 |  |  |
| Yes | 1.9 | 1.33-2.70 |  | 1.71 | 1.18-2.49 | 0.005 |
| **Postoperative chemotherapy** |  |  | 0.779 |  |  |  |
| No | 1 |  |  |  |  |  |
| Yes | 0.95 | 0.65-1.38 |  |  |  |  |
| **Surgery type** |  |  | 0.065 |  |  |  |
| Hepatectomy | 1 |  |  | 1 |  |  |
| RFA | 1.36 | 0.94-1.97 | 0.099 | 1.02 | 0.61-1.71 | 0.935 |
| Hepatectomy+RFA | 1.64 | 1.04-2.61 | 0.035 | 1.24 | 0.73-2.09 | 0.429 |
| **KRAS mutation** |  |  | 0.033 |  |  |  |
| No | 1 |  |  | 1 |  |  |
| Yes | 1.64 | 1.04-2.58 | 0.034 | 2.18 | 1.35-3.52 | 0.002 |
| unknown | 0.93 | 0.64-1.36 | 0.719 | 1.25 | 0.83-1.88 | 0.279 |
| **BRAF mutation** |  |  | 0.158 |  |  |  |
| No | 1 |  |  |  |  |  |
| Yes | 3.16 | 0.75-13.2 | 0.115 |  |  |  |
| unknown | 0.86 | 0.62-1.19 | 0.356 |  |  |  |
| **Hepatic steatosis** |  |  | 0.006 |  |  |  |
| Without | 1 |  |  | 1 |  |  |
| With | 1.74 | 1.18-2.59 |  | 1.86 | 1.23-2.82 | 0.003 |
