# Supplementary material for: Hepatic Steatosis Predicts Higher Incidence of Recurrence in Colorectal Cancer Liver Metastasis Patients
Source: Front Oncol. 2021 Mar 9;11:631943. doi: 10.3389/fonc.2021.631943 (PMC7986714; doi:10.3389/fonc.2021.631943)
Supplement: Supplementary Table 2 — Risk factors for hepatic RFS by univariate and multivariate Cox regression analysis. [file Table_2.DOCX]

**Table S2. Risk factors for hepatic RFS by univariate and multivariate analysis**

|  | **Univariate analysis** | | | **Multivariate analysis** | | |
| --- | --- | --- | --- | --- | --- | --- |
| **Variables** | **HR** | **95% CIs** | **P** | **HR** | **95% CIs** | **P** |
| **Total** |  |  |  |  |  |  |
| **Age at diagnosis** |  |  | 0.971 |  |  |  |
| ≤60 | 1 |  |  |  |  |  |
| >60 | 1.007 | 0.70-1.45 |  |  |  |  |
| **Sex** |  |  | 0.819 |  |  |  |
| Male | 1 |  |  |  |  |  |
| Female | 0.95 | 0.64-1.42 |  |  |  |  |
| **BMI** |  |  | 0.579 |  |  |  |
| ≤25 | 1 |  |  |  |  |  |
| >25 | 1.13 | 0.73-1.77 |  |  |  |  |
| **Diabetes Mellitus** |  |  | 0.828 |  |  |  |
| Without | 1 |  |  |  |  |  |
| With | 1.06 | 0.62-1.83 |  |  |  |  |
| **Primary tumor location** |  |  | 0.09 |  |  |  |
| Colon | 1 |  |  |  |  |  |
| Rectum | 0.71 | 0.47-1.06 |  |  |  |  |
| **Depth of tumor invasion** |  |  | 0.017 |  |  |  |
| ≤T3 | 1 |  |  | 1 |  |  |
| T4 | 1.47 | 1.08-2.27 |  | 1.37 | 0.94-2.00 | 0.099 |
| **Lymph node stage** |  |  | 0.461 |  |  |  |
| N0 | 1 |  |  |  |  |  |
| N1 | 1.09 | 0.87-1.37 |  |  |  |  |
| N2 |  |  |  |  |  |  |
| **Maximum size of liver metastases** |  |  | 0.357 |  |  |  |
| ≤5 cm | 1 |  |  |  |  |  |
| >5 cm | 1.24 | 0.79-1.94 |  |  |  |  |
| **Number of liver metastasis** |  |  | <0.001 |  |  |  |
| ≤5 | 1 |  |  | 1 |  |  |
| >5 | 3.52 | 2.13-5.83 |  | 2.99 | 1.73-5.18 | <0.001 |
| **Preoperative chemotherapy** |  |  | <0.001 |  |  |  |
| No | 1 |  |  | 1 |  |  |
| Yes | 2.13 | 1.43-3.19 |  | 1.85 | 1.21-2.83 | 0.004 |
| **Postoperative chemotherapy** |  |  | 0.338 |  |  |  |
| No | 1 |  |  |  |  |  |
| Yes | 0.82 | 0.55-1.23 |  |  |  |  |
| **Surgery type** |  |  | 0.1 |  |  |  |
| Hepatectomy | 1 |  |  |  |  |  |
| RFA | 1.44 | 0.96-2.16 | 0.078 |  |  |  |
| Hepatectomy+RFA | 1.58 | 0.95-2.66 | 0.081 |  |  |  |
| **KRAS mutation** |  |  | 0.021 |  |  |  |
| No | 1 |  |  | 1 |  |  |
| Yes | 1.67 | 1.02-2.72 | 0.041 | 2.44 | 1.46-4.09 | 0.001 |
| unknown | 0.86 | 0.57-1.30 | 0.466 | 1.28 | 0.83-2.00 | 0.266 |
| **BRAF mutation** |  |  | 0.092 |  |  |  |
| No | 1 |  |  |  |  |  |
| Yes | 3.28 | 0.78-13.77 | 0.105 |  |  |  |
| unknown | 0.79 | 0.55-1.14 | 0.204 |  |  |  |
| **Hepatic steatosis** |  |  | 0.002 |  |  |  |
| Without | 1 |  |  | 1 |  |  |
| With | 1.95 | 1.28-2.96 |  | 2.07 | 1.33-3.22 | 0.001 |
