# Supplementary material for: Hepatic Steatosis Predicts Higher Incidence of Recurrence in Colorectal Cancer Liver Metastasis Patients
Source: Front Oncol. 2021 Mar 9;11:631943. doi: 10.3389/fonc.2021.631943 (PMC7986714; doi:10.3389/fonc.2021.631943)
Supplement: Supplementary Table 3 — Scale for quality assessment of included studies for meta-analysis. [file Table_3.docx]

| Table S3.Scale for quality assessment |  |
| --- | --- |
| Criteria | Score |
| Representativeness of cases |  |
| Consecutive/randomly recruitment from case population | 2 |
| No method of selection stated | 0 |
| Ascertainment of colorectal cancer |  |
| Histological conﬁrmation at the Department of Pathology | 2 |
| Not described | 0 |
| Representativeness of controls |  |
| Consecutive/randomly recruitment from the same sampling | 1 |
| No method of selection stated | 0 |
| Diagnosis of liver disease |  |
| Histological analysis | 2 |
| Others | 0 |
| Sample size |  |
| ≥800 | 1 |
| <800 | 0 |
| Follow-up |  |
| Describe follow-up time | 1 |
| Describe how many patients were lost to follow up or not available for statistical analysis | 1 |

Total 10
